# Selenium for the Prevention of Cutaneous Melanoma

**DOI:** 10.3390/nu5030725

**Published:** 2013-03-07

**Authors:** Pamela B. Cassidy, Heidi D. Fain, James P. Cassidy, Sally M. Tran, Philip J. Moos, Kenneth M. Boucher, Russell Gerads, Scott R. Florell, Douglas Grossman, Sancy A. Leachman

**Affiliations:** 1 Department of Medicinal Chemistry, Huntsman Cancer Institute, 2000 Circle of Hope, Salt Lake City, UT 84112, USA; 2 Department of Dermatology, Huntsman Cancer Institute, 2000 Circle of Hope, Salt Lake City, UT 84112, USA; E-Mails: heididfain@hotmail.com (H.D.F.); cassidyutah@comcast.net (J.P.C.); doug.grossman@hci.utah.edu (D.G.); sancy.leachman@hci.utah.edu (S.A.L.); 3 University of Utah School of Medicine, 50 North Campus Dr., Salt Lake City, UT 84112, USA; E-Mail: sally.tran@hsc.utah.edu; 4 Department of Pharmacology and Toxicology, University of Utah, Salt Lake City, UT 84112, USA; E-Mail: philip.moos@pharm.utah.edu; 5 Biostatistics Unit, Huntsman Cancer Institute, 2000 Circle of Hope, Salt Lake City, UT 84112, USA; E-Mail: kenneth.boucher@hci.utah.edu; 6 Applied Speciation, 18804 Northcreek Parkway, Bothell, WA 98011, USA; E-Mail: russ@appliedspeciation.com; 7 Department of Dermatology, University of Utah School of Medicine, 50 North Campus Dr., Salt Lake City, UT 84112, USA; E-Mail: scott.florell@hsc.utah.edu; 8 Department of Oncological Sciences, Huntsman Cancer Institute, 2000 Circle of Hope, Salt Lake City, UT 84112, USA

**Keywords:** selenium, melanoma, selenomethionine, methylseleninic acid, HGF mouse

## Abstract

The role of selenium (Se) supplementation in cancer prevention is controversial; effects often depend on the nutritional status of the subject and on the chemical form in which Se is provided. We used a combination of *in vitro* and *in vivo* models to study two unique therapeutic windows for intervention in the process of cutaneous melanomagenisis, and to examine the utility of two different chemical forms of Se for prevention and treatment of melanoma. We studied the effects of Se *in vitro* on UV-induced oxidative stress in melanocytes, and on apoptosis and cell cycle progression in melanoma cells. *In vivo*, we used the HGF transgenic mouse model of UV-induced melanoma to demonstrate that topical treatment with l-selenomethionine results in a significant delay in the time required for UV-induced melanoma development, but also increases the rate of growth of those tumors once they appear. In a second mouse model, we found that oral administration of high dose methylseleninic acid significantly decreases the size of human melanoma xenografts. Our findings suggest that modestly elevation of selenium levels in the skin might risk acceleration of growth of incipient tumors. Additionally, certain Se compounds administered at very high doses could have utility for the treatment of fully-malignant tumors or prevention of recurrence.

## 1. Introduction

Dietary selenium (Se) deficiency is associated with increased risk for heart disease, immune dysfunction, male infertility and cancer [1]. Se is an essential micronutrient incorporated into proteins in the form of the amino acid selenocysteine. There are 25 selenoproteins in humans (24 in the mouse) and several of these, including three different thioredoxin reductases (TRs), four GPxs and selenoprotein P are believed to detoxify reactive oxygen species (ROS) [2]. The roles of selenoproteins in the skin are not well characterized, although GPx2 [3] and GPx4 [4] are thought to be important regulators of redox homeostasis in this tissue. TR1 may be important as well since it supplies reduced thioredoxin to the peroxiredoxins, which are antioxidant proteins highly expressed throughout the skin [5]. 

In a review of the literature published by researchers at the Fred Hutchinson Cancer Center, 64%–80% of cancer patients reported using vitamin and mineralsupplements; and of these, approximately 30% contain Se and other antioxidants [6]. This is a matter of significant concern to clinicians who treat melanoma patients since little is known about the effects of Se supplements on the risk for melanoma or on its treatment. Interest in the use of Se for the prevention of cancer dates back to the early 1990s when Blot *et al.* reported that a vitamin supplement which included Se significantly decreased mortality (principally due to lower rates of stomach cancer) for study participants in Linxian, China, a region where dietary Se deficiency was common [7]. In the United States, the Nutritional Prevention of Cancer Study Group conducted a trial designed to test the utility of Se as a single agent for the prevention of non-melanoma skin cancer (NMSC) in 1996. While they found no effect on the incidence of skin cancers, patients receiving Se in the form of selenized yeast had a reduced risk for total cancer as well as site specific cancers of the prostate, lung and colon [8]. These promising results for Se and other antioxidants [9] as potential chemoprevention agents inspired the trial of Se and vitamin E for the prevention of prostate cancer (SELECT), a study that enrolled more than 35,000 men [10]. The study was discontinued in 2009 after a finding of no protection from Se; additional follow-up of study participants demonstrated a statistically significant increased risk for prostate cancer in the vitamin E arm [11]. Because of these and other negative results from trials of antioxidants for cancer prevention [12], leaders in the field have called for more rigorous pre-clinical testing of new agents. Their recommendations include conducting pre-clinical studies of disease-specific mechanisms of action that include the identification of intermediate biomarkers of efficacy, testing of agents in appropriate animal models, and in the case of Se-containing agents, careful consideration of the specific chemical forms of Se used with respect to their differential activities and metabolisms in humans [13,14,15]. In this work we incorporate these post-SELECT recommendations in cell culture and animal studies that examine the utility of Se as an agent for the prevention and treatment of melanoma. 

Ultraviolet (UV) radiation from sunlight is the primary environmental factor linked to melanoma risk. However the classic “UV signature” mutations in tumor suppressor genes arising from the formation of intra-strand pyridine dimers in NMSC [16] are not the only mutations commonly found in melanomas [17,18,19]. Meyskens and Fisher have proposed that melanoma development is mediated at least in part by oxidative damage to DNA [20,21]. ROS, induced by both UV radiation and as a bi-product of pigment biosynthesis, cause the formation of a number of oxidative lesions in DNA, which if not removed prior to DNA replication, can result in mutations [22]. Damage to DNA by ROS is likely facilitated by the depletion of antioxidant defenses such as the glutathione peroxidases (GPxs) and the small molecule antioxidant glutathione (GSH) in UV-irradiated skin [23,24]. Our own results support an important role for oxidative stress in melanoma; we found that the prodrug N-acetylcysteine delays the onset of UV-induced melanoma in the hepatocyte growth factor (HGF) transgenic mouse model (also used in this work) when administered prior-to and shortly after UV-irradiation [24]. 

**Figure 1:**
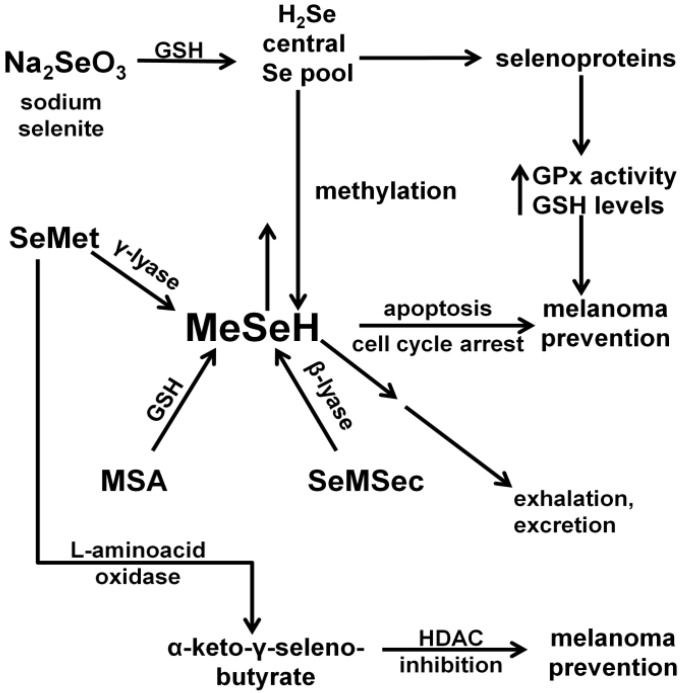
Metabolism of various chemical forms of Se and their chemopreventive activities.

Because many of the selenoproteins function as antioxidants, we reasoned that maximizing the activities of these proteins by topical treatment with supplemental selenomethionine (SeMet) might prevent melanoma, especially at the initiation stage [25] when normal melanocytes (the cells from which melanomas arise) in the skin are under severe genotoxic and oxidative stress. However, SeMet has another potentially beneficial effect mediated by its low molecular weight metabolite methyl selenol (MeSeH, Figure 1) [26]. This species has been found in other cancer models to be toxic to initiated cells (those having one or more mutations but not fully transformed) or early-stage cancers, at concentrations having little or no effect on normal cells [27]. This activity could be exploited for melanoma prevention at the promotion stage. Our experimental design allows us to examine the potential role(s) of both selenoproteins and MeSeH at the initiation and progression stages of melanomagenisis in the HGF transgenic mouse model of UV-induced melanoma. We further explore the utility of MeSeH as a cancer therapy in a xenograft model of melanoma. 

As shown in Figure 1, Selenite is reduced by GSH and enters the central selenium pool in the form of hydrogen selenide (**H_2_Se**). From there it can be utilized in the synthesis of selenocysteine and incorporated into antioxidant selenoproteins such the GPxs and the TRs. A single methyl group can be added to Se from the central pool to give **MeSeH**, which can induce apoptosis and/or cell cycle arrest. MeSeH can also be methylated a second time giving dimethyl selenide, which is exhaled. A third methylation, giving trimethylselenonium ion or alternatively incorporation of Se into a selenosugar, results in forms that are excreted in the urine or feces. **SeMet** enters the central selenium pool by way of the γ-lyase catalyzed generation of MeSeH. MeSeH then enters the central Se pool by demethylation. Additionally, L-amino acid oxidase can deaminate **SeMet** to give α-keto-γ-selenobutyrate, an HDAC inhibitor with potential melanoma prevention activity. **MSA** and methylselenocysteine (**SeMSec**) can be transformed to MeSeH by reaction with GSH and enzyme catalyzed β-lyase reactions, respectively.

## 2. Experimental Section

### 2.1. Se Compounds and Other Reagents

Sodium selenite, SeMSec and MSA were purchased from Sigma Aldrich (St. Louis, MO, USA). Cell culture supplies were from Life Technologies (Grand Island, NY, USA). Bid antibody was from R&D Systems (Minneapolis, MN, USA). The Bip/GRP78 antibody was purchased from BD Biosciences (San Jose, CA, USA). Anti-SPARC (human osteonectin) was from Novacastra (Newcastle upon Tyne, UK). Anti-elf2α and anti-phospho-elf2α (Ser51) were from Cell Signaling Technologies (Beverly, MA, USA). Anti-α-tubulin and HRP-conjugated secondary antibodies were from Santa Cruz Biotechnology (Santa Cruz, CA, USA). Proteins were visualized with ECL plus reagent (GE Healthcare, Piscataway, NJ, USA). Matrix metalloprotease inhibitor GM-6001 was purchased from EMD Chemicals (Gibbstown, NJ, USA).

### 2.2. Cell Culture

SK-Mel 28 (derived from human primary cutaneous melanoma), and HTB-66 (human metastatic melanoma) were purchased from American Type Cell Culture and were cultured according to the supplier’s recommendation in the presence of 50 units/mL penicillin and streptomycin. Lox and Yusac2 cell lines (both metastatic human melanomas) were a gift from Dr. Ruth Halaban (Yale University) [28] and were maintained in Dulbecco’s modified Eagle’s medium (DMEM) plus antibiotics supplemented with fetal calf serum (5% (v/v) for Yusac2 and 10% for Lox). The Melan-a mouse melanocyte cell line, derived from C57/BL/6 mice, was kindly provided by Dorothy Bennett (St. George’s University of London, London, United Kingdom) [29] and maintained in RPMI 1640 containing 10% FCS, 200 nmol/L phorbol 12-myristate 13-acetate, 200 pmol/L cholera toxin, and antibiotics.

### 2.3. XBP1 Splicing Analysis

Total RNA was isolated with the TRIzol reagent (Life Technologies Inc.) and was purified using RNeasy kit (Qiagen, Valencia, CA, USA). First strand cDNA was synthesized from 100 ng of total RNA by SuperScript II reverse transcriptase (Invitrogen, Carlsbad, CA, USA) following the manufacturer’s protocol. The XBP1-specific primers were those reported by Zu *et al.* [30]. The amplified fragment was digested with Pst I and the resulting products were resolved on a 2% agarose gel containing ethidium bromide and photographed using UV illumination.

### 2.4. Normal Human Melanocytes

Melanocytes were isolated from neonatal foreskins as described [31]. Early passage cells were treated with gentamycin in order to eliminate any contaminating fibroblasts.

### 2.5. UV Irradiation

A bank of four fan-cooled unfiltered sun lamps (FS20T12-UVB, National Biological Corp.) emitting 4 W/m^2^ was used for all experiments. These bulbs emit wavelengths between 250 and 420 nm (72.6% UVB, 27.4% UVA, and 0.01% UVC), with peak emission at 313 nm, according to the manufacturer. Dosimetry was monitored using a UVB-500C meter (National Biological).

### 2.6. GPx Activity Assay

Cells and tissues were lysed in a buffer containing 50 mM Tris pH 7.5, 100 mM NaCl, 1 mM DTT. Total GPx activity was measured as described by Brigelius-Flohe *et al.* using t-butyl hydroperoxide as substrate [32].

### 2.7. Glutathione Measurement

Reduced glutathione was measured as described elsewhere [33] after derivitization with monobromobimane using an HPLC assay with fluorescence detection. Protein was determined using the BCA method (Pierce, Milwaukee, WI, USA). 

### 2.8. Cell Cycle Analysis

After treatments, cells were trypsinized, washed with PBS, resuspended in Nuclear Isolation and Staining Solution (NIM-DAPI, Beckman Coulter Miami, FL, USA), and incubated for 1 h in the dark. Samples were analyzed using flow cytometry (Cell Lab Quanta SC, Beckman Coulter) with a minimum of 20,000 events recorded for each sample. Cell cycle distributions were estimated using ModFit LT software Version 2.0.

### 2.9. Cell Viability Assay

Cells were seeded into 96-well tissue culture plates and after overnight incubation, treated with MSA. Cell viability was determined 48 h after treatment using the tetrazolium-dye based assay CellTiter Aqueous One Solution Proliferation Assay (Promega, Madison, WI, USA). 

### 2.10. Caspase Assay

Caspase-3 activity in cell lysates was assessed using the fluorescent substrate DEVD-AFC (R&D Systems) according to the protocol reported previously [34].

### 2.11. Topical SeMet Treatment

A lotion containing SeMet (0.02% l-selenomethionine w/w) was prepared from 1.0 mL phosphate-buffered saline (PBS) containing 2 mg l-selenomethionine (Aldrich) incorporated into 9.0 g Dermabase^®^ (Paddock Labs, Minneapolis, MN, USA). The control lotion contained PBS alone in the same proportion. 

### 2.12. Total Se Quantification of Tissue Samples by ICP-DRC-MS

Prior to analysis, all samples were digested using concentrated HNO_3_. An aliquot of each sample was weighed into Teflon vials followed by the addition of concentrated HNO_3_. The vessels were immediately capped and heated until the solid material fully dissolved. The digested samples were analyzed by inductively coupled plasma dynamic reaction cell mass spectrometry (ICP-DRC-MS) at Applied Speciation, Bothell, WA, USA.

### 2.13. Animals

Animals were handled according to protocols approved by the University of Utah Institutional Animal Care and Use Committee. 

***HGF Transgenic Mice***—HGF-Tg (BL6 MH19) mice on a C57/Bl/6 background [35] were provided by Glenn Merlino (National Cancer Institute). Transgenic males were mated to wild-type C57/BL/6 females (Charles River, Wilmington, MA, USA). Approximately 24 h after birth, animals were treated with topical lotions (20 mg, 1.6 μg Se). After an additional 24 h, pups expressing HGF (indicated by chocolate-point phenotype) were placed in uncovered 6-well cell culture plates, each well containing a strip of double-sticky tape which prevented the animals from turning on to their backs. UV radiation (4000 J/m^2^) was then administered using the apparatus described above. The animals were returned to their mothers and were left undisturbed until weaning at 3 weeks of age. Thereafter they received topical treatments (100 mg, 8.0 μg Se) twice weekly and were shaved and their skin examined for tumors once per week. At 23–25 weeks of age, the animals were sacrificed and tissue (tumors, lymph nodes, normal skin) were collected. 

***Animal Feed***—For the preliminary tests of Se in the skin of neonatal mice, the high Se diet was Harlan Teklad (Indianapolis, IN, USA) torula yeast-based diet TD.06534 Se Supplemented Diet (2.25) with 2.25 ppm Se as sodium selenite. The control diet was Harlan Teklad torula yeast-based diet TD.06533 Selenium Control Diet (0.3) with 0.3 ppm Se. For the melanoma prevention study, mating pairs and pregnant and nursing females were fed a custom high fat diet (Teklad 3080). Weaned animals were fed a standard rodent diet (Teklad 8656). All diets for the prevention study contained 0.35 ppm Se.

***Xenograft Studies***—Male Nod/SCID mice bred at the University of Utah were provided by Alana Welm. Mice 6–9 weeks of age were injected with 1 million Lox cells in 0.20 mL McCoy’s 5A medium as reported [36]. Treatment with MSA is described in the text. After two weeks, tumors were excised, weighed and analyzed. 

### 2.14. Statistical Analysis

Cox Proportional Hazards Model was used to analyze tumor data in the prevention study with 31 animals in the control arm and 40 animals in the SeMet arm. All other statistical comparisons were performed using two-tailed Student’s *t*-tests. Were indicated, error bars represent standard deviation.

The software package used for analysis was R Development Core Team (2011) R: A Language and Environment for Statistical Computing, R Foundation for Statistical Computing, Vienna, Austria [37]. 

### 2.15. Immunochemical Analysis (Western Blot)

Cells and tissues were lysed or disrupted in buffer (0.05 M Tris pH 7.4, 0.1 M NaCl, 2 mM EDTA, 0.1% SDS, 0.1% deoxycholate, 1 mM NaF, 1 mM sodium orthovanadate with Sigma protease inhibitor cocktail) at 4 °C. After protein estimation (Coumassie Plus (Bradford) Protein Assay, Pierce (Thermofisher), Rockford, IL, USA) equal portions were loaded onto polyacrylamide gels, and the separated proteins were transferred to PVDF membranes and analyzed using HRP-conjugated secondary antibodies with luminescence detection.

## 3. Results

### 3.1. Se Restores Selenoprotein Activity and GSH Levels in UV-Irradiated Melanocytes

**Figure 2:**
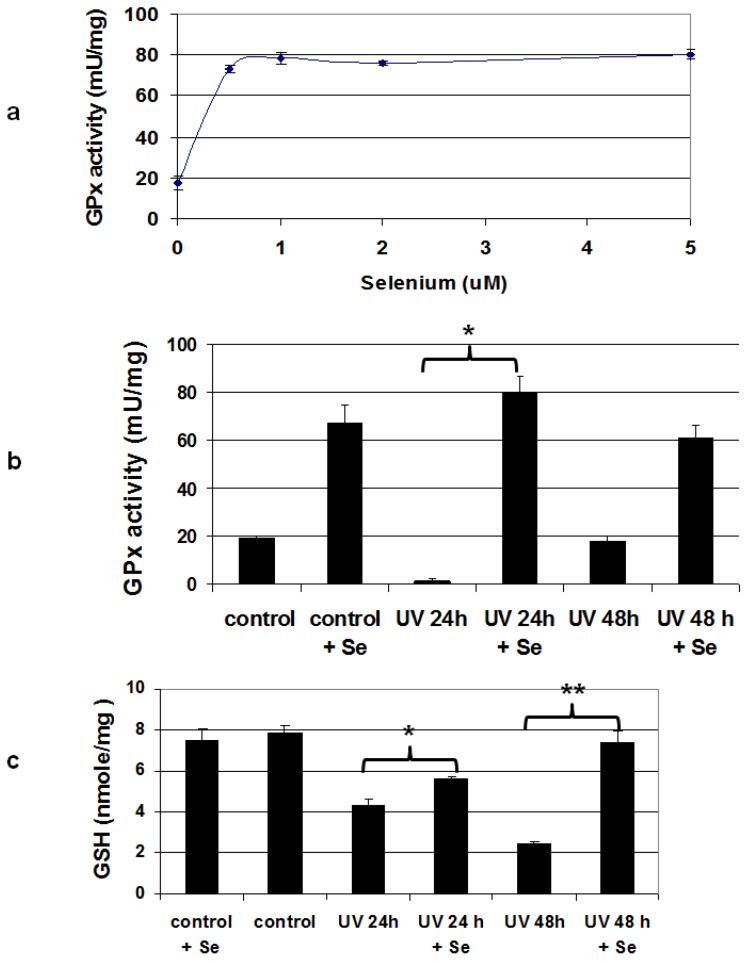
Se supplementation restores antioxidant activity in UV-irradiated mouse melanocytes. (**a**) Treatment with sodium selenite for 24 h increases GPx activity in un-irradiated cells by more than 4.6-fold compared to those grown in basal medium, *n* = 3 for each point. (**b**) UV radiation (1000 J/m^2^) severely depletes GPx activity in irradiated melanocytes 24 h after irradiation, but pre-treatment with 500 nM sodium selenite restores enzyme activity to that of controls (* *p* ≤ 0.008), *n* = 3 for each condition. (**c**) Melanocytes irradiated with UV (1000 J/ m^2^) had significantly reduced levels of glutathione at 24 and 48 h after irradiation. Glutathione in cells pre-treated for 24 h with 500 nM sodium selenite was higher in irradiated cells at 24 h post-irradiation, and was restored to levels of untreated controls after 48 h (* *p* < 0.01; ** *p* = 0.023).

Sodium selenite (Na_2_SeO_3_) is the preferred chemical form of Se for maximizing selenoprotein activity in cell culture because it does not require enzymatic release of Se (a property absent in many cell lines) before entry into the central Se pool (Figure 1 and [38]), and it is not toxic at levels required for supplementing selenoprotein activity [39]. Many cell culture medium formulations are Se-deficient and do not support maximal selenoprotein activity [40], and we found that this is also true with our basal medium used for the mouse melanocyte cell line Melan-a. GPx activity, which is a standard metric of Se sufficiency, is increased significantly by Se supplementation and is maximal in Melan-a cells after addition of 500 nM selenite (Figure 2a). We also found a more modest increase in the activity of TR after addition of supplemental Se (Figure S1, [41]). UV radiation causes a significant reduction in the antioxidant defenses of the skin, and this is manifested both by a decrease in GPx activity and depletion of GSH in human epidermis [23]. Since <10% of cells in the epidermis are melanocytes, we determined whether UV had the same effects on pure melanocyte cultures (Figure 2b,c). We observed a dramatic UV-induced decrease in GPx activity in Melan-a cells at 24 h post-irradiation, though the activity recovered at 48 h. GPx activity depletion was completely relieved by pre-treatment with Se; cells treated with both Se and UV had GPx activity indistinguishable from cells treated with Se alone at both 24 and 48 h. We also saw a decrease in GSH levels in melanocytes at both 24 and 48 h after irradiation (Figure 2c). This effect was partially ameliorated at the 24 h time point by addition of 500 nM sodium selenite prior to treatment with UV; by 48 h GSH levels in the irradiated cells treated with Se had rebounded to that of the unirradiated controls. 

### 3.2. Topical SeMet Delays Onset of UV-Induced Melanoma

**Figure 3:**
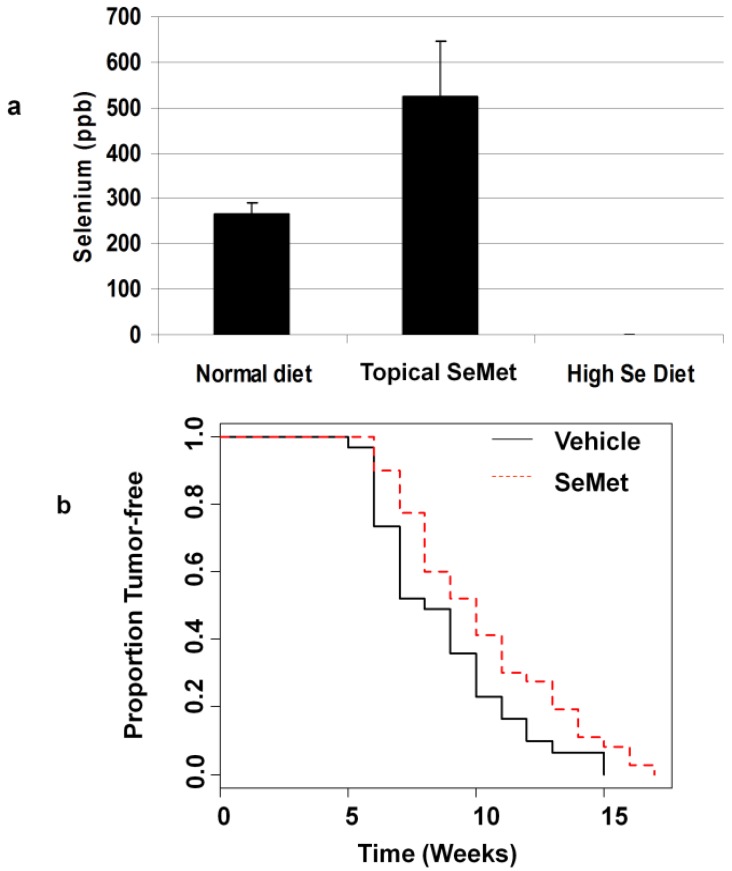
Topical selenium increases selenium levels in neonatal mouse skin after a single dose and delays formation of UV-induced melanomas. (**a**) Application of topical SeMet 24 h after birth increases total selenium in the skin of neonatal mice (*p* = 0.024 for normal diet *vs.* topical SeMet, *n* = 3 for each treatment). Supplementing the diet of the mother decreases selenium in the skin of the offspring to undetectable levels (High Se diet). (**b**) SeMet applied 24 h before UV irradiation (4000 J/m^2^) [24] then twice weekly after weaning significantly increases the time for tumor formation in 50% of animals (10 weeks *vs.* 8 weeks, *p* = 0.036), *n* = 31 for control, *n* = 40 for SeMet.

To determine the efficacy of Se as a melanoma prevention agent *in vivo*, we used a mouse model in which animals are engineered to express HGF in the skin [35]. In the HGF model, optimal induction of melanoma occurs when animals are treated with UV radiation 1–2 days after birth. This requirement was a significant issue with our experimental design in that we needed a treatment regimen that would elevate Se in the skin of neonates at the time of irradiation. We tried both an oral supplement (2.25 ppm Se in the feed of the mother, supplied as sodium selenite in a torula yeast-based feed) and a topical lotion containing selenomethionine (SeMet) which was applied 24 h before UV treatment. We based these treatments on that used in a previous study of SeMet for prevention of squamous cell carcinoma [42]. In our study, the topical treatment was also effective; a single application increased Se concentrations in neonates from 272 ppb in the controls, to 515 ppb in animals receiving topical Se (Figure 3a). The Burke study found that adult mice fed a high Se diet had Se in the skin at levels twice those of controls; however we did not see the same effect on Se in the skin of neonates in our study. High Se in the feed of the mothers resulted paradoxically, in a level of Se in the skin of the neonates that was below our level of detection (less than 0.1 ppb, Figure 3a). 

In the prevention trial animals were treated topically with either vehicle or SeMet-containing lotion one day after birth, and then irradiated on the following day. All mice were given a standard diet containing 0.3 ppm Se [43]. Treatment with the topical agent resumed with twice weekly applications after weaning at 3 weeks of age. Tumor size and number were scored weekly. We found that the SeMet-treated animals developed tumors later than the vehicle-treated animals (10 weeks *vs.* 8 weeks for control, *p* = 0.034, Figure 3b). We also determined the Se levels in the skin and found that the SeMet treated animals had Se levels 2.3-fold higher than controls (847 ppb *vs.* 365 ppb, *p* = 0.007). There were no differences in size, number of tumors or tumor histology at the end of the study (24 weeks). Inguinal and axillary lymph nodes in both groups were grossly pigmented and histologically demonstrated small clusters of pigmented epithelioid cells within the lymph node parenchyma and subcapsular sinuses; however definitive metastatic foci were not observed (Figure S2). In all animals examined, liver spleen and lungs were grossly normal in appearance. 

We also examined the dynamics of tumor growth and development taking into account treatment group and sex of the animals (see Figure S3 for graphical representation of the data). Cox proportional hazards model for time to first tumor indicates that the SeMet group had lower risk than controls. Males had slightly higher risk, although this was not statistically significant (Table 1). Multiple linear regression analysis of tumor count and area showed a significant increase in the rate of increase of both metrics after 7 weeks in the SeMet-treated group. The rate of increase in tumor number in males was also slightly higher (Table 2).

**Table 1:** Cox proportional hazards model for time to first tumor indicates that the SeMet group had lower risk than controls. Males had slightly higher risk, although this was not statistically significant. A goodness of fit test using only the treatment factor (SeMet or Control using the cox.zph function) gives *p* = 0.68 indicating no violation to the proportional hazards assumption.

Predictor	Coefficient	Hazard Ratio	Lower 95% CI	Upper 95% CI	*Z*	*p*-value
Group = SeMet	−0.5182	0.5956	0.3668	0.9672	−2.095	0.036
Sex = m	0.2364	1.2667	0.7840	2.0464	0.966	0.33

**Table 2:** Multiple linear regression analysis shows that tumors number and area grow more rapidly in SeMet treated animals than in controls. For tumor number, exponential growth rate in for each animal after 7 weeks is the response variable. For tumor area tumor quadratic growth rate after 7 weeks for each animal is the response variable. In each, the untreated female controls are the reference group.

**Tumor number: **Multiple Linear Regression of Slopes for Individual Lines				
**Parameter**	**Estimate**	**SE**	***t***	***p*-value**
(Intercept)	0.15196	0.03326	4.57	2.05 × 10^−5^
SeMet	0.08455	0.03421	2.472	0.0159
Male	−0.06901	0.03431	−2.01	0.0482
**Tumor area: **Multiple Linear Regression of Slopes for Individual Lines				
**Parameter**	**Estimate**	**SE**	***t***	***p*-value**
(Intercept)	0.27541	0.03819	7.212	5.13 × 10^−10^
SeMet	0.08307	0.03928	2.115	0.038
Male	−0.05537	0.03940	−1.405	0.164

SeMet can release MeSeH in a γ-lyase-catalyzed reaction (Figure 1). This metabolite has chemopreventive properties of its own [16], but can also enter the central Se pool where it is available for incorporation into selenoproteins. In order measure the effects of our topical SeMet treatment on selenoprotein activity, we measured GPx activity and GSH levels in the skin of neonates. Animals were irradiated with UV (4000 J/m^2^) after topical treatment with either SeMet containing cream or vehicle control. GPx activity in the skin was high (200 mU/mg), and there was no difference in the dorsal skin of animals treated with UV and either topical SeMet or vehicle control. We saw a slight decrease in GSH in the skin of animals treated with UV and vehicle control compared to those treated with vehicle alone at 24 h after irradiation, but this difference was not statistically significant (Figure S4, [37]). 

### 3.3. The Small Molecule Metabolite MeSeH Causes Cell Cycle Arrest and Induces Apoptosis in Melanoma Cells

**Figure 4:**
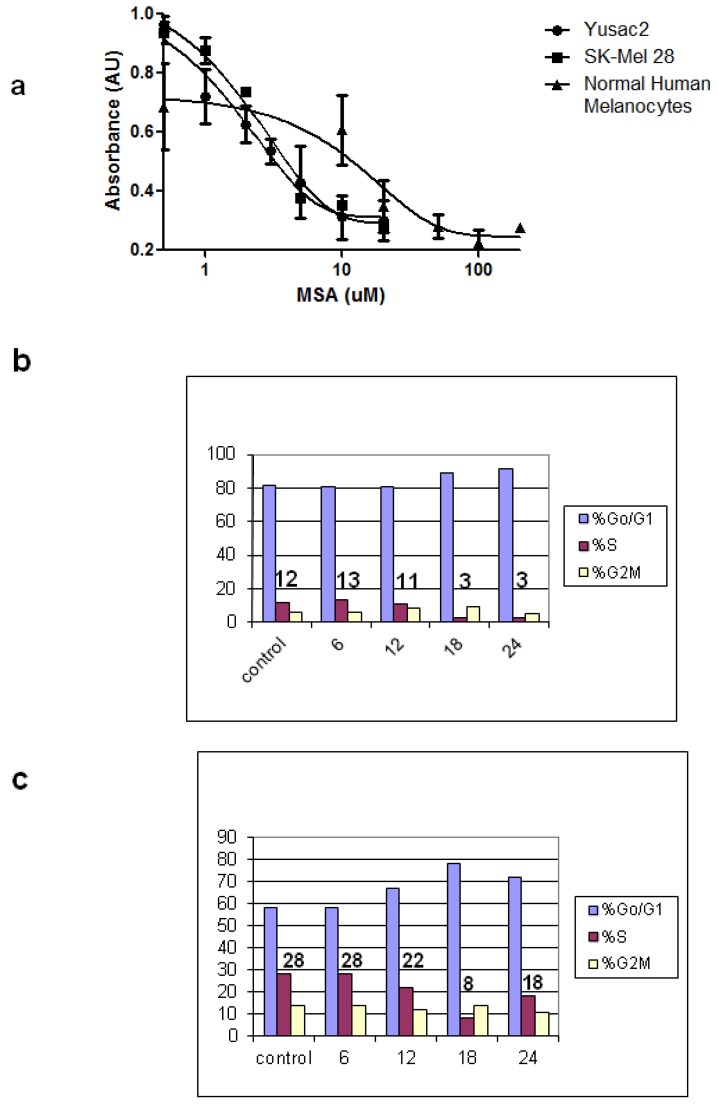
MSA inhibits cell growth and causes cell cycle arrest in human melanoma cells. (**a**) The MeSeH prodrug MSA inhibits the growth of melanoma cells (SK-Mel 28 and Yusac2) with IC_50_s of 2–3 µM, while normal human melanocytes are much less sensitive (IC_50_ = 20 μM) *n* = 3 for each data point. Treatment of SK-Mel 28 (**b**) and Yusac2 cells (**c**) with 15 μM MSA causes the cells to arrest in the G_0_/G_1_-phase. Numbers above bar graphs indicate % of cells in S-phase. Results are representative of 2 separate experiments.

MeSeH is produced in biological systems from a variety of precursors as illustrated in Figure 1. In cell culture, MeSeH is conveniently generated by treatment of cells with methylseleninic acid (MSA) [26]. MeSeH is released from MSA by non-enzymatic reaction with cellular GSH (Figure 1). We treated two melanoma cell lines as well as normal human melanocytes with increasing concentrations of MSA and measured the effects on cell viability with a tetrazolium dye-based assay (Figure 4a). We found that the IC50s for both SK-Mel 28 and Yusac2 melanoma cell lines was almost 10-fold lower than that of human melanocytes (2–3 μM for melanomas, 20 μM for melanocytes). Next, we examined the effects of MSA on cell cycle progression in two melanoma cell lines. We found both SK-Mel 28 and Yusac2 cells accumulated in the G_0_/G_1_ phases after treatment with 15 µM MSA, and the Yusac2 cells began to recover after 24 h (Figure 4b,c). 

### 3.4. The Unfolded Protein Response (UPR) is Induced by MSA

**Figure 5:**
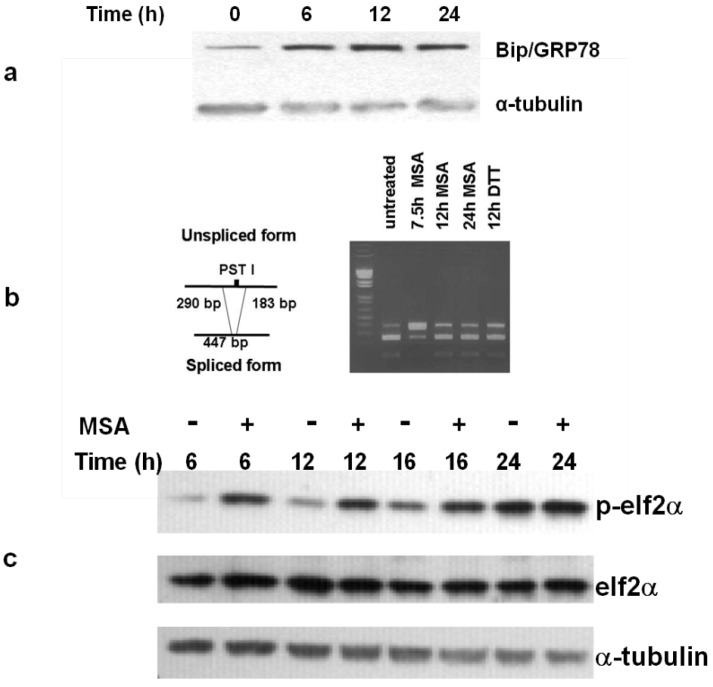
MSA induces the UPR in human melanoma. (**a**) MSA treatment (15 μM) initiates the unfolded protein response in SK-Mel 28 melanoma cells as shown by increased levels of Bip/GRP78. (**b)** MSA activates IRE1 as assessed by analysis of XBP1 cDNA. In the mature message produced by IRE1 the PST 1 cleavage site is spliced out giving the 447 bp product. (**c**) elF2α is phosphorylated by release of PERK from the ER of melanoma cells treated with 15 μM MSA for the indicated times. Results are representative of 2 separate experiments.

MSA treatment induces endoplasmic reticulum (ER) stress which elicits the UPR in prostate cancer cells [30]. One component of this phenomenon is the increase in protein levels of the molecular chaperone Bip/Grp78 which we observed in SK-Mel 28 melanoma cells treated with MSA (Figure 5a). We surveyed 5 additional melanoma cell lines (Yusac2, WM 793, Lox, Yugen 8 and HTB66) and found that they all reacted similarly (data not shown). We examined other aspects of the UPR including phosphorylation of eIF2α, activation of IRE1 (as assessed by splicing of mRNA XBP1) and ATF6 cleavage (as assessed by changes in protein levels of CHOP/Gadd153). Melanoma cells responded as anticipated with respect to the first two parameters (Figure 5b,c), but unlike prostate cancer cells did not show any change in protein levels for CHOP/Gadd 153 (data not shown). Reports from the Hersey lab [44], corroborate our results. They found that untreated melanoma cells express a relatively high level of CHOP/Gadd153 that actually decreased slowly over time after treatment with well-characterized inducers of the UPR, thapsigargin and tunicamycin.

### 3.5. MSA Induces Activation of Caspases

**Figure 6:**
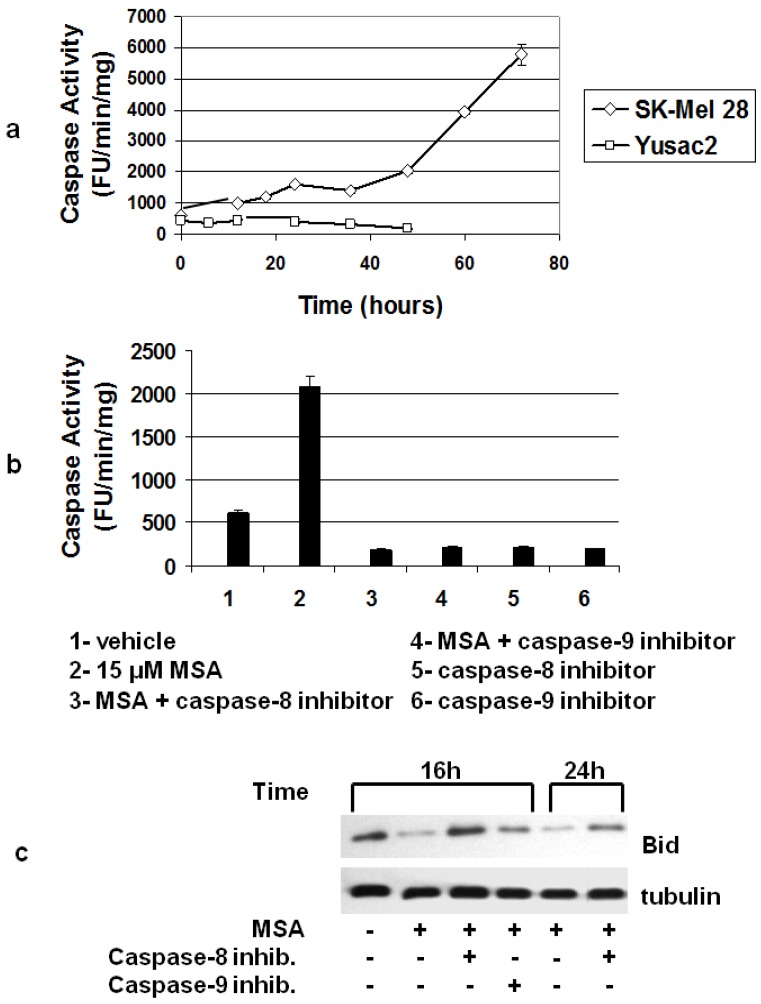
MSA activates caspase- 3 via caspases -8 and -9 in a subset of melanoma cells. (**a**) MSA treatment (15 μM) activates caspase-3 in SK-Mel 28 melanoma cells, but not in Yusac2 cells, *n* = 3 for each data point. (**b**) Both caspase-8 and -9 inhibitors block activation of caspase-3 in HTB66 cells treated for 24 h with 15 μM MSA, *n* = 3 for each data point. (**c**) MSA decreases full-length Bid in melanoma cells. Bid levels are restored by both caspase-8 and -9 inhibitors. Results are representative of 2 separate experiments.

In our exploration of MSA-induced activation of caspases, we found a time-dependent activation of caspase-3 after treatment of SK-Mel 28 cells with 15 μM MSA, but in Yusac2 cells, caspase-3 activity actually decreased over time (Figure 6a). We looked at caspase-3 activity in 3 other cell lines (Lox, Yugen8, HTB66) and found that 2 of these (Lox and HTB66) had increased caspase-3 activity after MSA treatment and Yugen8 did not (data not shown). Since HTB66 cells behaved similarly when treated with MSA and were easier to work with in culture (they grew much faster), we used this cell line in experiments where we investigated the mechanism of activation of caspase-3. In these experiments we treated cells with both MSA and inhibitors of initiator caspases -8 and -9 (Figure 6b). Both inhibitors eliminated MSA-induced caspase-3 activity. Together, the data in Figure 3b both fit a linear model where caspase-8 activation is upstream of both caspase-3 and caspase-9. A reasonable candidate for the mediator of caspase-8 activation of caspase-9 is Bid. Death receptor-dependent activation of caspase-8 has been shown to be associated with Bid cleavage into a smaller activated form [45]. The activated Bid (tBid) translocates to mitochondria and promotes cytochrome c release and activation of caspase-9. When we monitored Bid protein by immunohistochemical analysis we found that it was indeed cleaved after treatment of HTB66 cells with MSA, although our antibody reacted only weakly with the cleaved protein. However, the time course of disappearance of full-length Bid is consistent with its role as a mediator of caspase-3 activation and with the effects of specific caspase-8 and 9 inhibitors (Figure 6c).

### 3.6. MSA Decreases Levels of Secreted Proteins

**Figure 7:**
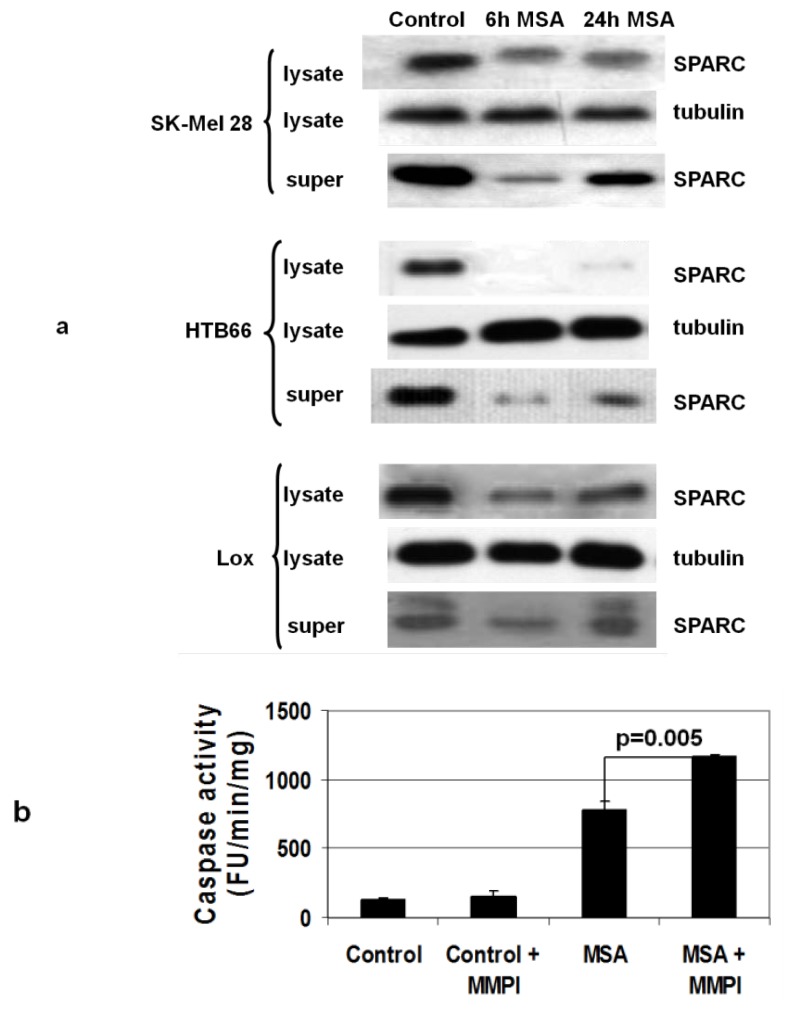
MSA decreases levels of secreted proteins in melanoma cells. (**a**) Treatment of melanoma cells with 15 μM MSA for the indicated times results in decreased SPARC levels in both lysates and medium. Results are representative of 3 experiments with each cell line. (**b**) A matrix metalloprotease inhibitor (MMPI) increases caspase-3 activation in HTB66 cells treated with MSA, *n* = 3 for each condition.

Excreted proteins undergo extensive processing in the ER and we reasoned that this class of proteins might be especially sensitive to the effects of ER stress. SPARC (secreted protein acidic and rich in cysteine) is found in the cell membrane and in medium of cultured melanoma cells [46], and downregulation of its expression in melanoma tumor xenografts by antisense RNA inhibits tumor growth [47]. We found that treatment of some melanoma cell lines (SK-Mel 28, HTB66 and Lox, (Figure 7a)) but not all (not Yusac2, Yugen 8 or WM793, data not shown) with MSA caused a decrease in SPARC both in cell lysates and in the medium. SPARC levels in the medium recover over time, perhaps as the methyl selenol produced by MSA treatment is methylated giving the inactive species dimethylselenide [48]. MSA-induced suppression of SPARC production correlates with caspase activation, *i.e.*, SK-Mel 28, HTB66 and Lox cells activate caspase-3, and Yusac2 and Yugen8 do not. Activation of matrix metalloproteinases (MMPs) by SPARC has been reported in other systems [49], and we investigate the possibility of such a connection in melanoma using a broad spectrum MMP inhibitor [50] on HTB66 cells treated with MSA. We saw an increase in caspase-3 activation in HTB66 cells treated with a combination of the drug and MSA compared to MSA alone, but no caspase activation with only the MMP inhibitor (Figure 7b). This is consistent with SPARC activation of an MMP which could in turn cleave and inactivate an as yet unidentified death receptor ligand that is responsible for the caspase-8 activity observed after treatment of melanoma cells with MSA.

### 3.7. MSA Decreases Growth of Human Melanoma Xenograft Tumors

**Figure 8:**
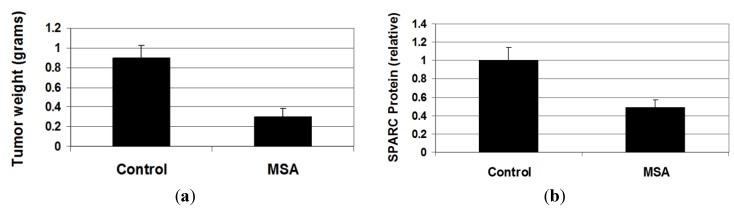
MSA decreases tumor growth of human melanoma *in vivo*. (**a**) Treatment of Nod/SCID mice bearing Lox melanoma xenograft tumors with oral MSA (1.25 mg Se/kg daily) decreases tumor weights (*p* = 0.0007) and (**b**) reduces SPARC levels in tumors (*p* = 0.067).

We next examined the effects of MSA on melanoma tumors in NOD/SCID mice. We chose to use a cell line from the class that showed both activation of caspases and suppression of SPARC excretion (Lox, SKMel-28 and HTB66) when treated with MSA. Of these, Lox cells were by far the most tumorogenic of the three cell lines in our hands, forming tumors in excess of 1 cm in diameter in two weeks after the subcutaneous injection of only 1 million cells. In these experiments, a solution of MSA (1.25 mg Se/kg in 30 μL sterile PBS) or PBS was given orally 3 h before injection of the melanoma cells and every 24 h thereafter for two weeks. The animals were then sacrificed and the tumors harvested. We found that MSA treatment reduced the weight of the tumors by more than 66% (*p* = 0.0007) (Figure 8a). There were no differences in body weight between the two groups (Figure S5). Immunochemical analysis of tumor lysates showed that SPARC levels in tumors from treated animals were lower than that in controls (Figure 8b; Figure S6, [51]), although the trend did not quite reach statistical significance. Caspase-3 cleavage was not evident by immunochemical analysis in either group (Figure S6, [51]). 

## 4. Discussion

The metabolic fate of Se is dependent on both its chemical composition and on dose [38,52], (Figure 1). As Se consumption rises to a level above that necessary for maintenance of selenoprotein activity (200–400 μg/day [1]), the profile of metabolites changes to include MeSeH. MeSeH is a small molecule that can be generated *in vivo* from a variety of forms of Se including SeMet and MSA. The effects of MeSeH (delivered by prodrugs) on cells in culture include cell cycle arrest, induction of apoptosis, and induction of the unfolded protein response. MeSeH prodrugs have also been found to be effective cancer prevention and treatment agents in both transgenic and xenograft models of human cancer [26,53,54]. Melanoma is the most dangerous form of skin cancer, and as a consequence there is considerable interest in developing agents for its prevention [55].

We began our studies of Se for prevention of melanoma at the initiation stage with UV-irradiated cultured melanocytes. We found that selenoprotein activity could be increased in untreated cells by adding Se, and that supplementation relieved both the UV-induced depletion of GSH and loss of GPx activity observed in cells grown in Se-deficient medium. We also found that treatment of melanoma cells with MSA (a prodrug form of MeSeH) caused cell cycle arrest and apoptosis in melanoma cell lines. These two potential protective activities at both the initiation and progression stages were the basis of the design of our melanoma prevention study with the HGF mouse, where we examined the efficacy of SeMet when applied both before and after mutagenic insult (UV irradiation). HGF mice have melanocytes situated in the dermis, at the epidermal/dermal junction and in the basal layer of the epidermis. In contrast, melanocytes in wild-type mice are located at the base of the hair follicle where they are unaffected by melanoma-initiating UV radiation. Melanocytes in HGF transgenic animals proliferate in response to UV radiation. A single neonatal dose of UV radiation causes melanomas that recapitulate many features of the human disease and provide an excellent model of human melanoma [35]. 

We chose SeMet for this study because of its efficacy in a mouse model of UV-induced squamous cell carcinoma (SCC, [42]). We based the post-initiation treatment schedule on that used in the Burke study where twice weekly applications of topical SeMet-containing lotions were shown to delay development of SCC. In our model, an additional application prior to UV treatment allowed us to examine the activity of this agent at the initiation stage as well. We found that when we analyzed the skin of the mice shortly after irradiation, GPx activity and GSH levels in the skin were not significantly different between animals pre-treated with Se or control lotions. This is in sharp contrast to our *in vitro* work where we found that Se supplementation significantly relieved UV-induced decreases in both of these antioxidant activities in melanocytes cultured in Se-deficient medium (Figure 2b,c). We surmise that Se supplied in the maternal diet was sufficient to maximize selenoprotein activity in the skin of the neonatal mice [56]. We could not confirm this by producing Se-deficient neonates because the minimum effect of a Se-deficient diet on breeding pairs would be a dramatic suppression of male fertility that would likely preclude reproduction. We must note that our model required a 3 week gap in treatment of the animals due to the sensitivity of the dams to handling of the pups. It is conceivable that SeMet treatment could have had an effect on phenomena occurring after our assessment of GPx activity and GSH levels, such as the immune response to UV; our model does not address this. Nevertheless, we believe that our experimental design provides results from *in vitro* and *in vivo* models which suggest that any protective effects of Se on melanoma at the initiation stage are likely to be important only in cases of Se deficiency, a condition easily remedied by a Se-sufficient diet. 

When we allowed tumors to develop in UV-irradiated animals, we found a statistically significant delay (10 weeks *vs.* 8 weeks) in the time required for appearance of tumors greater than 1 mm in 50% of animals treated with SeMet-containing lotion, compared to animals treated with vehicle alone. This delay in tumor formation is likely due to a preventive effect at the progression stage of melanomagenisis. The effect could be mediated by MeSeH, as the dose of SeMet (equivalent to 2 mg Se in a human) is likely sufficient to elicit the production of this metabolite [57]. Alternatively, therapeutic benefit could arise from generation of α-keto-γ-selenobutyrate, a SeMet metabolite recently shown to inhibit histone deacetylase activity in prostate cancer cells [58]. Chemical speciation of Se in the skin (which would be complicated by the volatility of MeSeH and dimethyl selenide) has not been performed to our knowledge. Therefore we cannot say with certainty which Se species is responsible for the delay in melanoma development that we observed, only that the effect was likely at a post-initiation stage.

Interestingly, when we analyzed tumor kinetics as measured both by tumor number and size, the rate of change in the SeMet group was actually higher than those of controls (Table 2). Thus we conclude that although SeMet delays tumor development to some extent, once tumors arise, they increase in number faster and grow at a higher rate. This has serious implications clinically, raising the possibility that application of SeMet to tissues harboring incipient microscopic tumors might actually accelerate tumor growth. This observation is consistent with the observation that moderately elevated levels of dietary selenium results in increased risk for liver cancer in a mouse model (insert reference here). We also looked at the effects of sex on risk for tumors and tumor dynamics. Males showed a slightly elevated risk (Table 1, not statistically significant) and slightly lower rate of increase in tumor number (Table 2).

In our work with the methyl selenol precursor MSA, we confirmed what was first reported by the laboratory of Clement Ip [30] for MSA-treated prostate cancer cells, that most features of the UPR were activated by MSA treatment in all of the melanoma cell lines that we examined. However when we measured caspase-3 activity in melanoma cell lines treated with MSA, 3 of the cell lines responded by activating this executioner caspase and 3 did not. We performed further analysis of caspase activation in the former class of cell lines using selective inhibitors of caspase-8 and caspase-9, along with immunochemical analysis of the cleavage of Bid. We concluded that activation of caspase-3 must arise from caspase-8 mediated cleavage of tBid which then translocates to the mitochondrion resulting in the activation of caspase-9. 

Convinced that MSA-induced apoptosis required activation of the extrinsic pathway for apoptosis, we began to look for effects of MSA on death receptors and molecules that regulate their activity. Inhibition of MMP activity in melanoma cells stabilizes death receptors including TNF-RI, DR4 and Fas [59]. In lung adenocarcinoma cells MMP-2 siRNA induces Fas-mediated activation of caspase-8 and -9 and cleavage of Bid by increasing Fas and FasL in both in the cell membrane and in the medium [60]. Also, tunicamycin, an inducer of the UPR, sensitizes melanoma cells to TRAIL-induced apoptosis by increasing levels of the death receptor DR5 [44]. In our SK-Mel 28 and HTB66 cells, we saw modest evidence of an increase in the expression of DR5 by RT-PCR, but immunochemical analysis did not reveal any significant increases in protein levels (data not shown). Since ER stress is known to affect the processing of excreted proteins, we also considered other proteins known to be produced by melanomas that might be involved in regulating death receptor activation. We found an excellent candidate in the protein SPARC, which is an activator of MMPs. Interestingly, we found SPARC to be secreted by all three of the cell lines that also activate caspase-3 when treated with MSA. We also found that treatment with a general MMP inhibitor (which had no effect as a single treatment) increased MSA-induced caspase-3 activity in melanoma cells. Although we have yet to identify the specific death receptors, ligands and MMPs involved, we are actively pursuing these leads as we believe that this information will lead to potentially useful combination treatments with MSA for melanoma.

Our *in vivo* studies resulted in a significant reduction in tumor burden in animals treated with MSA along with reduced levels of SPARC in the tumor tissue. These results are by themselves promising, but we hope that mechanistic studies will help us identify an agent that we can use in conjunction with MSA for total eradication of tumors. In preliminary cell culture studies we have examined other cytotoxic agents reported to have synergy with MSA in other tumor types (taxol, *cis*-platin) as well as DTIC which is approved for treatment of metastatic melanoma, but we have not yet found a combination with increased cytotoxicity in melanoma cells. We are continuing our search for synergistic combinations looking at more targeted agents as well as siRNAs.

The first of two major obstacles to the use of Se for the prevention of melanoma and other cancers are the largely disappointing results obtained thus far for chemoprevention trials using different forms of Se at supranutritional levels. Marshall *et al.*, begin to address this issue in their study of the pharmacokinetics of the MeSH precursor selenomethylselenocysteine (SeMSec) in men [15]. They observed that the NPC trial (the successful trial upon which the SELECT trial was based) used selenized yeast as a Se source [21], and the SELECT trial used SeMet [22]. This is significant because while SeMet is the major Se-containing species in selenized yeast [61], it also contains SeMSec. SeMSec and MSA, both excellent sources of MeSeH, are highly effective at high doses in *in vivo* prevention models of prostate cancer [53,54]. Human studies, including that of Marshall (highest dose 1200 μg Se as SeMSec), and an earlier study of SeMet (highest dose 800 μg Se) [52], show that Se from SeMSec is cleared within 48 h from the plasma, likely due to its metabolism to the short-lived yet biologically active species MeSeH. In contrast, SeMet accumulates in virtually all proteins (a consequence of miss-incorporation in place of methionine), where it is largely unaffected by metabolic activities that might result in production of MeSeH. These results illustrate the vastly different pharmacokinetics of the Se in these two compounds, and highlight the fact that all organoselenium agents are not biologically equivalent sources of Se, and that they must be studied individually in the proper model systems before they are studied in patients.

The second major obstacle to the development of Se-containing agents for cancer prevention is potential toxicity. The highest dose of Se used in a controlled human study was 3.2 mg Se daily (orally) in the form of selenized yeast [61]. In this study, side-effects of Se consumption (brittle hair and nails, garlic breath) were reported by all participants, but no significant toxicities were observed. However, the dose of SeMSec necessary to achieve some of the dramatic effects observed in murine prostate cancer (3 mg/kg/day Se [52,53]) is equivalent to 17 mg of Se/day for a 70 kg human [62], or 170 times the amount required for a Se-replete human diet [63]. Preclinical toxicology studies of SeMSec conducted by the National Cancer Institute have found a no adverse effect level in both the rat and the dog equivalent to 5.7 mg Se (daily for 28 days) for a 70 kg human [64]. These results raise concerns about whether a dose 3 times higher, such as that required in prostate cancer prevention models, will be tolerable in humans. 

## 5. Conclusions

In our study, we applied SeMet topically, two times a week at a dose equivalent to 1.8 mg Se for a human. This method of administration (which could conceivably result in percutaneous absorption of Se as was observed in the mouse [42]) delivers a dose of Se that delays appearance of tumors in our *in vivo* model. However, our data also suggest that SeMet application may accelerate the growth of established tumors and this has serious implications for the use of nutritional supplements in melanoma patients. Additionally, our *in vitro* studies show that a much higher dose of the MeSeH prodrug MSA is selectively toxic to melanoma cells, and our *in vivo* results (with a dose equivalent to 6.9 mg Se/day for a 70 kg human) demonstrate decreased growth of xenografted tumors. These results make MSA an excellent candidate for use as an oral agent in the adjuvant setting, where prevention of recurrence or development of a second primary tumor is the therapeutic goal, and the tolerance for potential side-effects are higher [25]. However, the high dose required for the beneficial effects observed clearly moves MSA from the role of a nutritional selenium supplement to that of a drug, and before this or any Se agent can be proposed for clinical use, the Se specie(s) that elicit the protective effects reported and the mechanism by which they act must be unambiguously identified, and the pharmacokinetic behaviors and toxicities of proposed agents must be thoroughly characterized. We and others [65] are addressing these issues in ongoing studies.

## Supplementary Files

Supplementary File 1 Selenium for the Prevention of Cutaneous Melanoma (PDF, 392 KB)
