# Supplementary material for: Selenium for the Prevention of Cutaneous Melanoma
Source: Nutrients. 2013 Mar 7;5(3):725–49. doi: 10.3390/nu5030725 (PMC3705316; doi:10.3390/nu5030725)

Supplementary Data

Figure S1. Thioredoxin reductase activity in mouse mouse melanocyte culture is increased by addition of sodium selenite. Mouse melanocytes were incubated in medium described in the text to which was added sodium selenite at concentrations shown. After 24 h, thioredoxin reductase activity was mearsured in cell lysates.

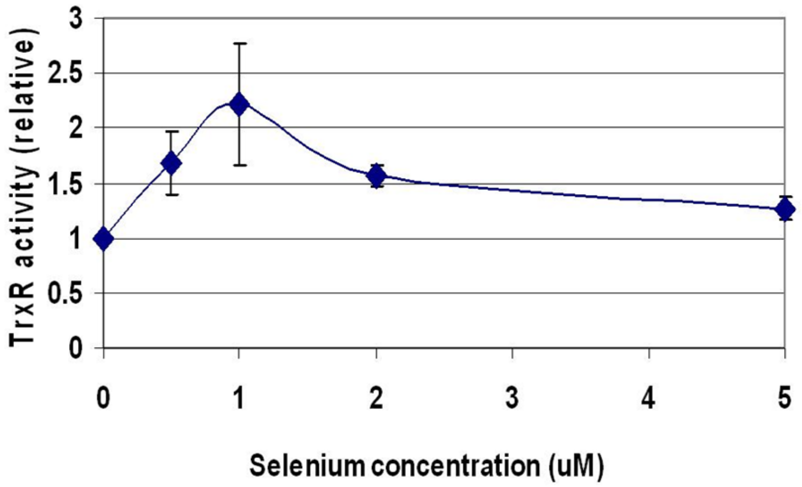

Figure S2. Tumors and lymph nodes from UV irradiated HGF mice. Both specimens are from mice treated with SeMet. Formalin-fixed paraffin-embedded tissue was stained with hematoxylin and eosin. Original magnification was 100×. (**a**) cutaneous melanoma, (**b**) inguinal lymph node.

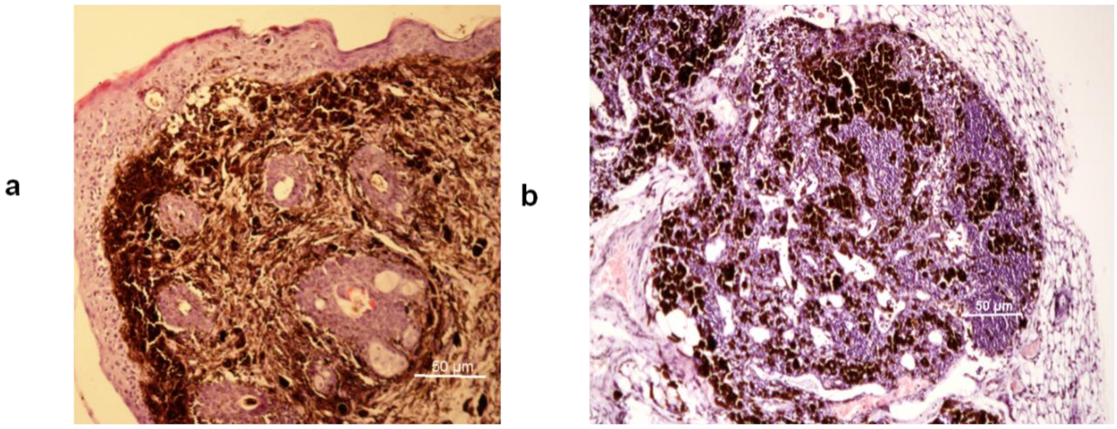

Figure S3. Plots of tumor count and area including line representing results of multiple linear regression analysis.

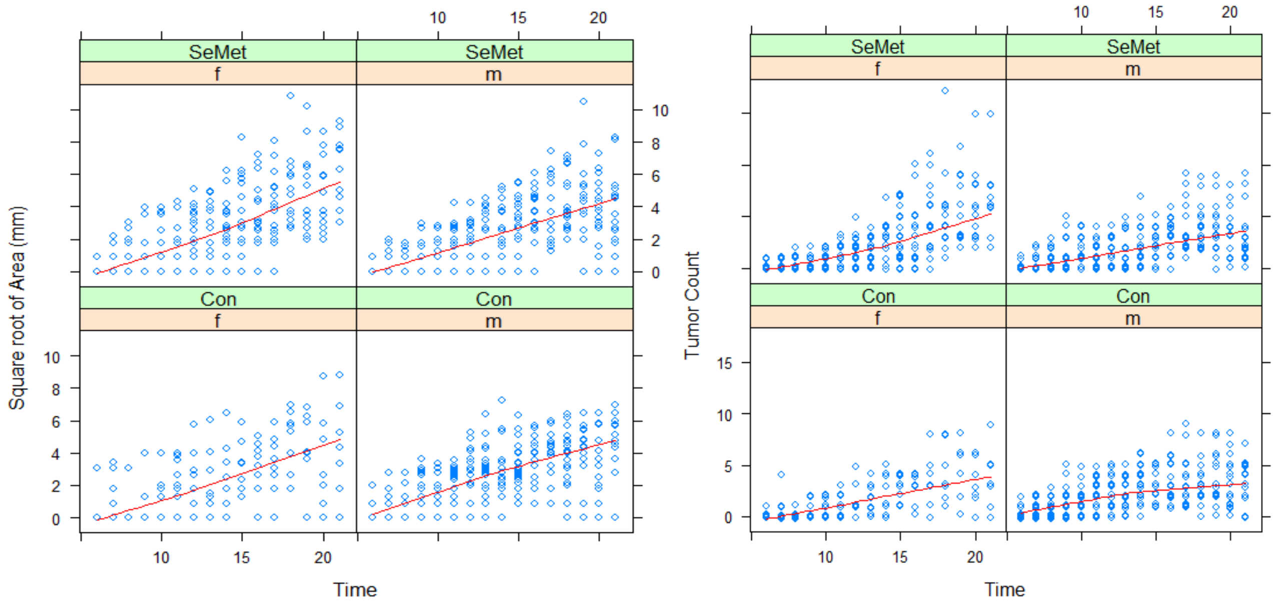

Figure S4. GSH in neonatal mouse skin. Vehicle and SeMet samples were treated with topical lotions then irradiated (4000 J/m^2). Dorsal skin was harvested after the indicated times. Two animals were used in each group. A mixed ANOVA model was used to analyze the data at each time point, with one fixed (treatment) factor and one random (observational unit). The factor was coded so that "vehicle" was the reference level. The estimate group effects are in comparison to "vehicle". The statistical analysis was performed using the "lme" function in the "nlme" package of "R" statistical computing software, version 2.8.0. The differences between Vehicle and SeMet and Vehicle and No UV skin at 24 h are not statistically significant (p = 0.21 and p = 0.16, respectively).

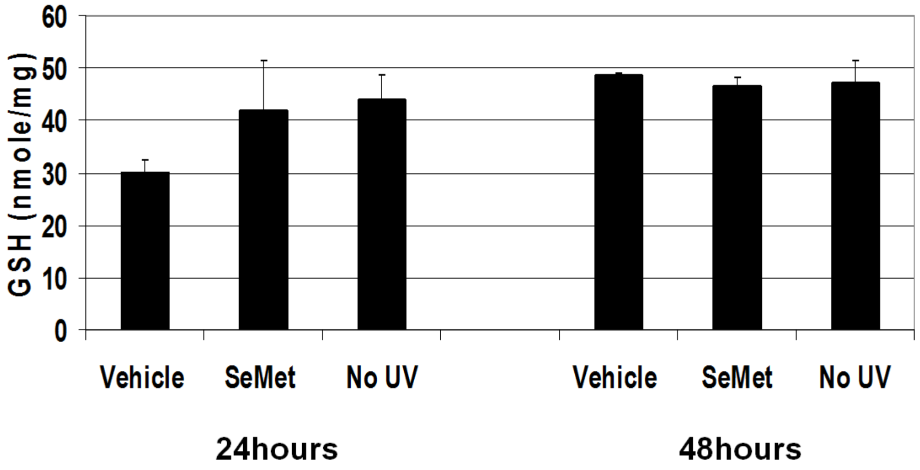

Figure S5. Oral treatment with MSA treatment does not affect weight of Nod-Scid mice. A solution of MSA (1.25 mg Se/kg in 30 μL sterile PBS) or PBS was given orally 3 h before injection of the melanoma cells and every 24 h thereafter for two weeks. The animals were then sacrificed and the tumors harvested. There were no differences in body weight between the two groups. Each point represents a single mouse. The average difference in weight (Day 14–Day 0) was −0.27 g for the control group and −0.09 g for the MSA group.

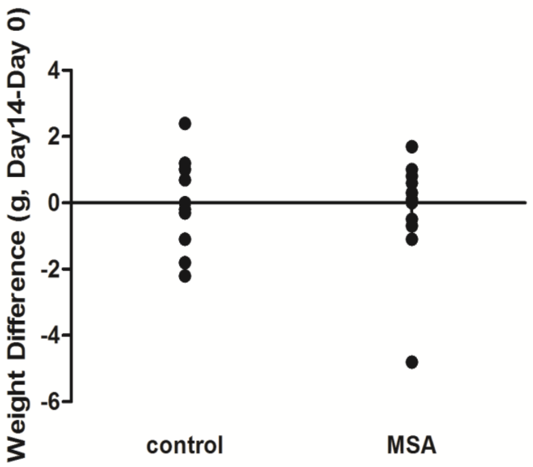

Figure S6. SPARC levels are decreased in tumors treated with MSA, but there is no evidence of increased activation of Caspase-3. Tumor tissue was lysed using the lysis buffer described in the methods section. Protein was loaded onto 10%–20% polyacrylamide gels and transferred to PVDF membrane. Mouse IgG heavy chain is visible on both the α-SPARC and α-tubulin blots; these antibodies are both mouse monoclonals and the upperbands likely arise from the mouse. All three images are from the same membrane, stripped after each analysis. **U** = vehicle controls **T** = MSA-treated. According to the manufacturer, the α-caspase-3 antibody should detect the cleaved (activated) peptide.

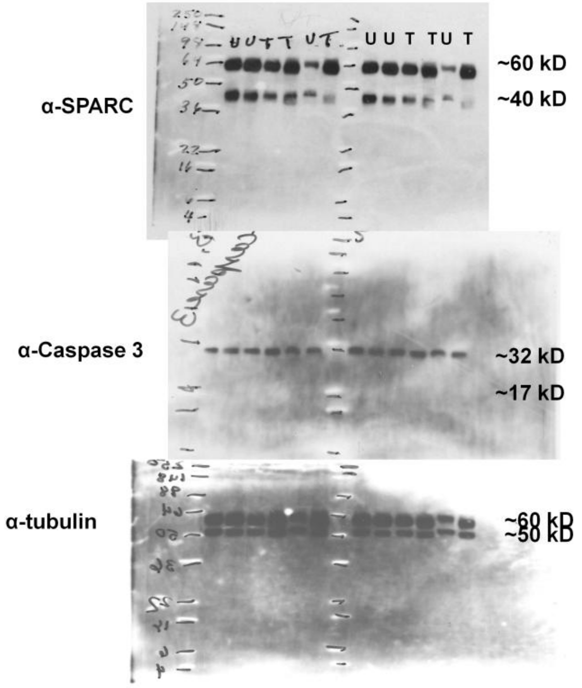

Supplement: Supplementary File 1 — Selenium for the Prevention of Cutaneous Melanoma (PDF, 392 KB) [file nutrients-05-00725-s001.pdf]
